# Patients’ tendency to recommend plastic surgery clinic shaped by appearance consciousness

**DOI:** 10.4102/hsag.v28i0.2320

**Published:** 2023-11-16

**Authors:** Nurliati S. Handini, Ferdi Antonio

**Affiliations:** 1 Department of Hospital Administration, Faculty of Economics and Business, Universitas Pelita Harapan, South Jakarta, Indonesia

**Keywords:** plastic surgery, patient experience, recommendation likelihood, postoperative patient consciousness of appearance

## Abstract

**Background:**

Plastic surgery services need adjustment from a patient experience perspective. However, its association with outcome quality was rarely studied. Postoperative appearance can play the role in predicting the clinic recommendations likelihood.

**Aim:**

This study is aimed to analyse recommendation likelihood (LRC) to the clinic, incorporating postoperative patient consciousness of appearance (PCA) as a mediator.

**Setting:**

Postoperative patient from two plastic surgery clinics in Jakarta, the Capital of Indonesia.

**Methods:**

Quantitative study with postoperative patient self-reported survey and cross-sectional data from 97 respondents were taken purposively. Respondent data were analysed through partial least squares structural equation modelling (PLS-SEM).

**Results:**

Five elements of patient experience had positive association with LRC (*p* < 0.05) mediated by PCA, while PCA demonstrated a large effect on LRC (β: 0.403; *p* = 0.004; confidence interval [CI] = 0.173–0.671). Thus, PCA can mediate the relationship of patient experience to encourage clinic recommendations. Furthermore, healthcare by plastic surgeons showed predominant relationships followed by staff service and accessibility, suggesting those should be of utmost concern to plastic surgery patients.

**Conclusion:**

Patient experience elements have positive association with LRC mediated by PCA. Therefore, the importance of PCA as a quality outcome must be considered in quality care delivery at plastic surgery clinics. Clinic management should prioritise optimising factors contributing to PCA.

**Contribution:**

This study showed evidence that postoperative consciousness of appearance as outcome quality occurred as a mediator in patient experience relationship towards behavioural intention.

## Introduction

The number of patients undergoing plastic surgery related to one’s appearance is increasing rapidly. There were 10 129 528 aesthetic plastic surgery procedures done globally according to a survey on aesthetic or cosmetic procedures in 2020 (International Society of Aesthetic Plastic Surgery 2020). The top five procedures were breast augmentation, liposuction, eyelid surgery, rhinoplasty and abdominoplasty. Apart from being in a hospital, there are also many consultations, examinations and certain treatments for patients carried out in private clinics which offers greater comfort for the patient. The competition among healthcare service providers such as clinics has greatly increased due to the expansion of the healthcare sector (Strumann et al. 2022).

The statistical numbers show that plastic surgery procedures are still in high demand, despite the COVID-19 pandemic. The pandemic situation coerced the plastic surgery services to be adjusted due to increased health and safety precautions (Saggaf & Anastakis 2021). Interestingly, the plastic surgery services were able to be maintained against all odds. Nevertheless, with more and more new plastic surgery clinics being opened, the competition is getting tougher; therefore, the clinic management needs to rethink its strategy. One approach is to use the recommendations voiced by former patients who received care at the clinic. Recommendations like this are more trusted and considered genuine because they come from the patient’s own experience (Marsidi, Maurice & Luijendijk 2014).

The common physical beauty perceived mostly arises from facial appearance as well as the perception of body image. The concept of beauty keeps evolving and continuously being a challenge for plastic, reconstructive and aesthetic surgeons across the globe (Chen et al. 2018). In general, the art of plastic surgery is consistently directed towards the normalisation of appearance and function (Coady 1997). Patients who suffer from disfigurements and deformities are seeking for approximation of normal appearance, while those who are in a healthy condition are seeking better harmony from an aesthetic perspective (Cano, Klassen & Pusic 2019; Harris & Carr 2001). The interventions done through plastic reconstructive and aesthetic surgery procedures are giving a relief of psychological distress and improvement in social as well as psychological functioning.

In an ideal setting, physicians deliver evidence-based care aligned with the preferences of patients, thereby improving satisfaction and efficient use of the resource (Coady 1997). However, patients may have the tendency to be more or less satisfied with distinct care-seeking patterns and have limitations to evaluate the technical quality, thus focusing more on the functional aspects such as the caring attitude and facility (Endeshaw 2021). This applies evidently in plastic surgery where the patients tend to have high expectations (Cano et al. 2019). Research by Fenton et al. (2012) resulted in an ambiguity in the understanding of what drives patient satisfaction or how it affects healthcare use and outcomes, as patients focus more on the hospitality attribute. Hence, it needs specific measures to ensure that care is evidence-based and patient-centred, in order to avoid overemphasis on patient satisfaction and unintentional on healthcare utilisation, cost and outcomes.

Objectively, a tool is needed to evaluate the outcome of such clinical procedures in healthcare service. In the field of plastic surgery, a so-called ‘perfect result’ from the surgeon’s perspective is not always synonymous with the patient’s assessment (Cano et al. 2019). A psychometric instrument to assess the specific problems of these patients is needed (Chen et al. 2018). Data acquired from patient-reported outcome (PRO) measures are essential both to healthcare quality and business performance (Addo, Mykletun & Olsen 2021; Pettersen 2004).

Patient-reported outcome measures are the instrument that quantifies the health-related quality of life and/or other significant outcome variables from the patient’s perspective and preference (Marsidi et al. 2014). A good PRO measure should be able to assess the impact of treatment or surgical intervention on various aspects of a patient’s outcome in a manner that is clinically meaningful, scientifically sound and practical (Addo et al. 2021; Cano, Browne & Lamping 2004). There were various instruments used but there is not yet sufficient evidence to use one instrument that is considered the most effective, especially in aesthetic plastic surgery patients in emerging countries such as Indonesia.

Previous research on patient satisfaction with plastic surgery showed questionnaire items, developed by Press Ganey, which could explain what factors were important to patients (Chen et al. 2018). Furthermore, that study also demonstrated factors correlated with the likelihood of recommending practice or provider. However, another study pointed out that in healthcare services, there are various quality aspects that patients will evaluate before behavioural intention (Swain & Kar 2018). One of the important aspects is the technical quality, where the patient will assess the outcome quality from his or her own perspective. Regarding plastic surgery service, the patient will judge the outcome from the results of the operation in the form of a change in his or her appearance (Harris & Carr 2001).

This study attempted to deploy the postoperative patient consciousness of appearance (PCA) as a predictor to recommend likelihood to the plastic surgery clinic while mediating the relation from patient experience. There were few self-reported measurements in plastic surgery postop patients’ evaluation (Chen et al. 2018; Marsidi et al. 2014; Reavey et al. 2011), one of them known as the Derriford Appearance Scale (DAS). The DAS59 focuses on the psychological factors related to postop patient appearance (Carr, Harris & James 2000). This measurement has been developed to meet the need for an objective measure of the spectrum of psychological conditions, including aesthetic problems and appearance (Carr et al. 2000; Harris & Carr 2001). This scale was developed for plastic surgery patients who have undergone several surgical procedures (e.g. abdominoplasty and breast surgery). This instrument offers benefits for patient selection in both aesthetic and reconstructive plastic surgery and in the evaluation of outcomes (Harris & Carr 2001). A further study (Cogliandro et al. 2016) developed an Italian version of DAS59 which proved to be a reliable method of assessing the appearance-related quality of life after plastic surgery procedures. There were also efforts to modify this instrument to the shorter form of 24 items, known as DAS24. Despite the pros and cons regarding DAS59 (Reavey et al. 2011), the concept of appearance from the patient perspective is worth to be considered in the technical quality evaluation of healthcare service in plastic surgery.

**FIGURE 1:**
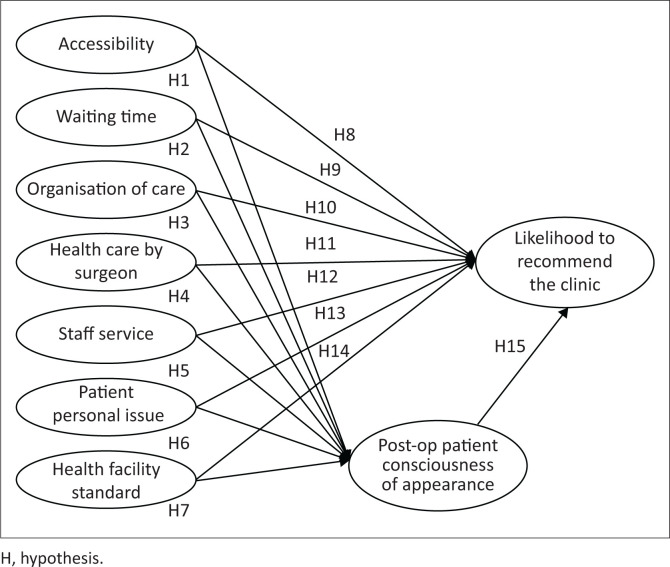
Conceptual framework.

This study contributes in two ways: firstly, providing a new point of view of having patients’ consciousness of appearance as an outcome quality that could mediate the element of patient experience in a plastic surgery setting. Secondly, this study highlighted elements of patient experience that occur and relate to the likelihood to recommend the aforementioned clinic. The conceptual framework of this study can be depicted in Figure 1.

The patient experience could be defined as the total sum of all interactions patients encounter in the healthcare treatment provided by healthcare professionals, staff and all the supporting facilities (Wolf et al. 2014). According to a previous study (Chen et al. 2018) regarding this experience, variables that were meticulously investigated consist of accessibility (ACS), waiting time (WTM), organisation of care (ORG), healthcare provided by the plastic surgeon (HCS), services from the clinic’s staff (SHS), personal issue of the patient (PPI) and the standards of the health facility (HFS) itself.

Previous studies demonstrated the importance of outcome quality from the service provider (Swain & Kar 2018). More recent research found that when consumer perceptions of experiential satisfaction increase, consumers will perceive better outcomes (Bellio & Buccoliero 2021). According to Reavey et al. (2011), patients have expectations for a better appearance as a result of plastic surgery. Carr et al. (2000) stated that appearance is a manifestation of what patients expect from plastic surgery services. Herewith, PCA can be considered as outcome quality, which may be applied in the context of the private clinic that provides plastic surgery services. Hence, the following hypotheses were proposed:

**H1:** Accessibility is associated positively with PCA

**H2:** Waiting time is associated positively with PCA

**H3:** Organisation of care is associated positively with PCA

**H4:** Healthcare by the surgeon is associated positively with PCA

**H5:** Staff service is associated positively with PCA

**H6:** Patient personal issue is associated positively with PCA

**H7:** Health facility standard is associated positively with PCA

Patient satisfaction has a positive impact on patient loyalty including willingness to recommend, according to an empirical study on healthcare services (Addo et al. 2021; Liu, et al. 2021; Yeo, Tan & Goh 2021). Another healthcare study in Indonesia also showed that there is a relationship between service antecedents of patient experience and loyalty. Previous studies done specifically in plastic surgery settings (Chen et al. 2018; Chung et al. 1999) revealed that the fulfilment of patient expectancy during the plastic surgery service was positively correlated to the likelihood to recommend the practice. From that previous research, it is known several factors from patient experience have a direct relationship with behavioural intentions. Therefore, the elements of patient experience could be implemented in the context of plastic surgery clinics. Hence, it can be hypothesised as follows:

**H8:** Accessibility is associated positively with LRC.

**H9:** Waiting time is associated positively with LRC.

**H10:** Organisation is associated positively with LRC.

**H11:** Healthcare by the surgeon is associated positively with LRC.

**H12:** Staff service is associated positively with LRC.

**H13:** Patient personal issue is associated positively with LRC.

**H14:** Health facility standard is associated positively with LRC.

Swain and Kar (2018) stated that technical quality is of high priority when it comes to patient satisfaction, indicated by clinical procedure and the quality of the outcome. In plastic surgery setting, the outcome is highly correlated with the individual perception of his or her appearance due to the surgery (Carr et al. 2000; Harris & Carr, 2001; Swami et al. 2007). Patients who experience the services and felt their appearance had changed according to their wishes will recommend the clinic to other potential future patients and thus it could be hypothesised as follows.

**H15:** Postoperative PCA is associated positively with the likelihood to recommend the clinic (LRC).

## Research methods and design

This study is designed using a quantitative patient self-reported survey approach with cross-sectional data. The objects in this study are all variables included in this research model. The dependent variable is the LRC, while accessibility, waiting time, organisation of care, healthcare by the surgeon, staff service, patient personal issue and health facility standards are the independent variables, and postop PCA acts as the mediating variable.

The questionnaire used in this research refers to relevant indicators from the previous study by Chen et al. (2018) and PCA scale (Harris & Carr 2001). All item indicators have gone through the process of face validity by a panel of five experts consisting of two healthcare management experts, a psychologist, a language expert and a field researcher. Prior to the face validity, the questionnaire was translated by linguists from English to Indonesian. In the face validity stage, 12 items were removed because they did not receive the above 80% agreement from the panel.

The authors collected the data purposively from 97 respondents at two plastic surgery clinics in Jakarta, Indonesia. All respondents had undergone plastic surgery procedures at those two clinics within the past year. Sampling was carried out in October 2022. G*Power (version 3.1.9.7) was used to estimate the required sample size (Memon et al. 2021) based on a significance level of 0.05, an effect size of 0.35 and a power of 0.80 for nine predictors. Accordingly, the calculated required minimum sample size for this study was 54. Questionnaires were distributed online to all the respondents referred to above with a response rate of 80%.

This study has an exploratory orientation, and the conceptual framework consists of nine constructs making it considered as a complex research model. Therefore, the partial least squares structural equation modelling (PLS-SEM) is suitable (Hair et al. 2019; Sarstedt et al. 2022). SmartPLS^®^ was a preferable software to analyse exploratory research; it was selected as it provides a bootstrapping menu to test significance (Memon et al. 2021).

The PLS-SEM procedures include measurement and structural models. The measurement model is established to measure the reliability and validity between indicators and their respective constructs in the model. The reliability testing phase includes indicator reliability (outer loading) and constructs reliability (Cronbach’s alpha and composite reliability). The validity testing phase includes construct validity through average variance extracted (AVE). After these requirements are met, then the structural model is then deployed to test the significant relationship between each construct in the research model (Sarstedt et al. 2022). Later, a mediation analysis and importance-performance map analysis (IPMA) were conducted as recommended (Hair et al. 2019).

Following the protection of human rights and welfare in research ethics, the researcher obtained ethical permission to collect data using a self-reported survey (001M/EC-10 January 2023). An informed consent form was made available as another measure to guarantee that this research did not go against ethical standards.

### Ethical considerations

This study has been approved by the Health Research Ethics Committee Universitas Pelita Harapan. 001M/EC-01 January 2023.

## Results

The respondents’ characteristics are represented in Table 1. Most of the respondents were female (84%, *n* = 52) and aged below 40 years (67%, *n* = 41). Patients came from private sector employees (51%, *n* = 32), and most of them are educated people who graduated from university. Data showed that most of the respondents visited the clinic 2–5 times within the past year, with their last visit within 1–3 months. This time span shows that respondents are still able to remember the health services they receive. Rhinoplasty and blepharoplasty are the two most commonly encountered types of surgery, and the patient mentioned plastic surgeon expertise as the reason for choosing the clinic.

**TABLE 1:** Respondents’ profile.

Description	Category	%	Sample (*n*)
Visit frequency to clinicwithin the past 1 year	Once	9	9
Visit frequency to clinicwithin the past 1 year Lastvisit to clinic	2–3 times	53	51
	4–5 times	22	21
	> 5 times	16	16
	Less than 1 month	22	21
Last visit to clinic Workstatus	1–3 months	47	46
	4–6 months	25	24
	7–12 months	6	6
	University student	5	5
Work status Education	Housewife	22	21
	Private sector employee	53	51
	Civil servant	5	5
	Entrepreneur	10	10
	Others	5	5
	High school graduate	12	12
Education Plastic surgery procedure	Graduate	86	83
	Postgraduate	2	2
	Blepharoplasty	21	20
Plastic surgery procedureReason of choosing clinic	Rhinoplasty	31	30
	Fat grafting	8	8
	Abdominoplasty	7	7
	Liposuction	13	13
	Breast augmentation	5	5
	Facelift	2	2
	Others	12	12
	Plastic surgeon’s expertise	77	75
Reason of choosing clinic	Privacy	9	9
	Location	6	6
	Facilities	5	5
	Cost	2	2

### Measurement model

The outer loading from the reflective model was done to assess the indicator of reliability. All indicators met the outer loading criteria, with a loading value above 0.708. In Table 2, the internal consistency reliability is satisfactory with Cronbach’s alpha greater than 0.7 and composite reliability ranging between 0.7 and 0.95; and acceptable convergent validity is shown with the AVE score above 0.5. (Hair et al. 2019). A list of questionnaire statements concerning the indicators can be seen in Appendix Table A1.

**TABLE 2:** Internal consistency reliability and convergent validity.

Variables	Cronbach’s alpha	Composite reliability	Average variance extracted (AVE)
Accessibility (ACS)	0.838	0.901	0.753
Waiting time (WTM)	0.089	0.948	0.901
Organisation of care (ORG)	0.904	0.094	0.084
Healthcare by plastic surgeon (HCS)	0.825	0.896	0.743
Staff service (SHS)	0.735	0.883	0.791
Patient personal issue (PPI)	0.084	0.904	0.076
Health facility standard (HFS)	0.911	0.938	0.791
Postoperative patient consciousness of appearance (PCA)	0.879	0.926	0.806
Likelihood to recommend clinic (LRC)	0.867	0.937	0.882

The discriminant validity of this research was assessed using a heterotrait-monotrait ratio (HTMT) method as recommended by Henseler, Ringle and Sarstedt (2015). The threshold of HTMT value is below 0.9, and the result shown in all the constructs has a HTMT ratio below 0.9. This step is crucial according to Hair et al. (2019) to confirm that each construct indicator is conceptually different. It is concluded that all indicators used in this research model have adequate discrimination to measure their respective constructs. Finally, this measurement model analysis has passed the parameters of all reliability and validity tests.

### Structural model

**FIGURE 2:**
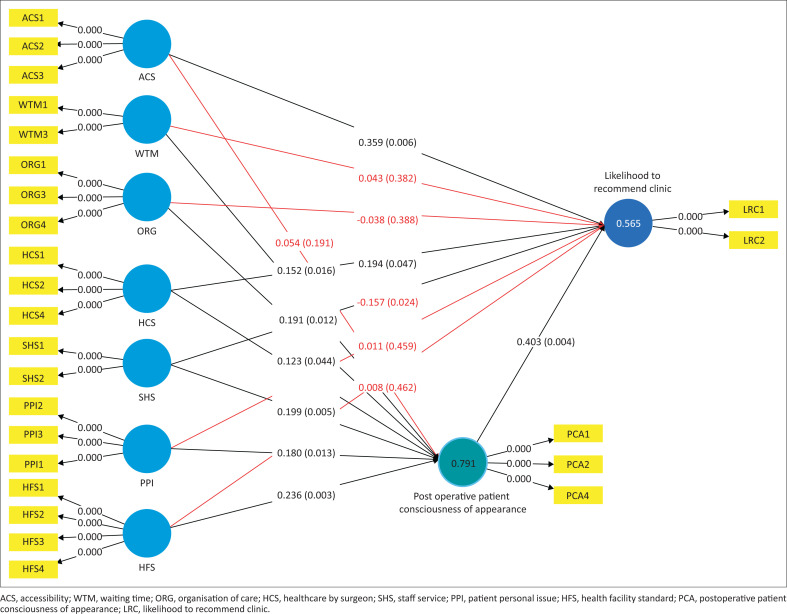
Partial least squares structural equation modelling structural model.

After the assessment of the measurement model, the further step was to assess the structural model which tests the relationship between each construct in the research model for its significance. The output of the PLS-SEM inner model through bootstrapping can be seen in Figure 2. Prior to that step, the quality of the model was also assessed. To that end, the inner variance inflation factor (inner VIF) was evaluated, and the result shows all the constructs had inner VIF under 5, which confirms no multicollinearity issue in this model.

In the structural model, the coefficient determinants or R^2^ were assessed to determine the prediction accuracy and explanatory power. The results found that PCA had an R^2^ of 0.791 and LRC had an R^2^ of 0.565 (Figure 2); both were categorised as moderate to strong estimation of accuracy (Hair et al. 2019). Both variables could be explained by more than 50% of the predictor variables in the model; thus, it could be said the model has an explanatory value. To establish an out-of-sample prediction approach, the Q^2^ predict was used as recommended by Shmueli et al. (2019). Based on the Q^2^ predict value, LRC has medium predictive relevance of 0.424, while PCA has large predictive relevance with a value of 0.758. Both R^2^ and Q^2^ assessments yielded acceptable results and thus it could be said that the model is adequate to predict behavioural intention.

Hypotheses testing by bootstrapping was conducted in order to determine the association of the variables in the model and confirm whether the proposed hypotheses were supported. The bootstrap, as a non-parametric approach, establishes the significance of the structural model through SmartPLS.4^®^ (Ringle, Wende & Becker 2022). The one-tailed test with an alpha 0.05 confidence interval (CI) of 5% and CI of 95% was used as a criterion to determine whether the hypothesis is significant (Hair et al. 2019; Sarstedt et al. 2022). The results are shown in Table 3.

**TABLE 3:** Hypotheses test result.

Hypothesis	Variables	*T*-statistics	CI 5%	CI 95%	Result
H1	ACS —> PCA	0.873	−0.045	0.158	Hypothesis not supported
H2	WTM —> PCA	2.134	0.040	0.270	Hypothesis supported
H3	ORG —> PCA	2.253	0.056	0.335	Hypothesis supported
H4	HCS —> PCA	1.707	0.004	0.241	Hypothesis supported
H5	SHS —> PCA	2.560	0.068	0.324	Hypothesis supported
H6	PPI —> PCA	2.232	0.041	0.305	Hypothesis supported
H7	HFS —> PCA	2.732	0.099	0.378	Hypothesis supported
H8	ACS —> LRC	2.536	0.108	0.574	Hypothesis supported
H9	WTM —> LRC	0.301	−0.185	0.287	Hypothesis not supported
H10	ORG —> LRC	0.283	−0.247	0.189	Hypothesis not supported
H11	HCS —> LRC	1.678	−0.001	0.378	Hypothesis supported
H12	SHS —> LRC	1.980	−0.288	−0.028	Hypothesis supported
H13	PPI —> LRC	0.102	−0.160	0.184	Hypothesis not supported
H14	HFS —> LRC	0.096	−0.120	0.163	Hypothesis not supported
H15	PCA —> LRC	2.688	0.173	0.671	Hypothesis supported

H, hypothesis; ACS, accessibility; WTM, waiting time; ORG, organisation of care; HCS, healthcare by surgeon; SHS, staff service; PPI, patient personal issue; HFS, health facility standard; PCA, postoperative patient consciousness of appearance; LRC, likelihood to recommend clinic.

It is listed in Table 4 that there are 7 hypotheses supported out of 15 hypotheses in the model with either positive or negative direction following the direction of hypotheses. From all the seven hypotheses that show the relationship between the elements of patience experience to PCA (H1–H7), only H1 was found to be not supported. Thus, accessibility (ACS) has no meaningful association with PCA. There are six contributing elements of patient experience impacting PCA, respectively, HFS, SHS, ORG, PPI, WTM and HCS, where HFS shows the greatest coefficient. This finding pointed out that health facility standard HFC is an important factor from patient perspective that should be considered.

**TABLE 4:** Significancy and coefficient.

Hypotheses	Variables	Standardised coefficient	*p* *	CI 5%	CI 95%	Result
H1	ACS —> PCA	0.054	0.191	−0.045	0.158	Hypothesisnot supported
H2	WTM —> PCA	0.152	0.016	0.004	0.027	Hypothesis supported
H3	ORG —> PCA	0.191	0.012	0.056	0.335	Hypothesis supported
H4	HCS —> PCA	0.123	0.044	0.004	0.241	Hypothesis supported
H5	SHS —> PCA	0.199	0.005	0.068	0.324	Hypothesis supported
H6	PPI —> PCA	0.018	0.013	0.041	0.305	Hypothesis supported
H7	HFS —> PCA	0.236	0.003	0.099	0.378	Hypothesis supported
H8	ACS —> LRC	0.359	0.006	0.108	0.574	Hypothesis supported
H9	WTM —> LRC	0.043	0.382	−0.185	0.287	Hypothesisnot supported
H10	ORG —> LRC	−0.038	0.388	−0.247	0.189	Hypothesisnot supported
H11	HCS —> LRC	0.194	0.047	−0.001	0.378	Hypothesisnot supported
H12	SHS —> LRC	−0.157	0.024	−0.288	−0.028	Hypothesisnot supported
H13	PPI —> LRC	0.011	0.459	−0.016	0.184	Hypothesisnot supported
H14	HFS —> LRC	0.008	0.462	−0.012	0.163	Hypothesisnot supported
H15	PCA —> LRC	0.403	0.004	0.173	0.671	Hypothesis supported

ACS, accessibility; WTM, waiting time; ORG, organisation of care; HCS, healthcare by plastic surgeon; SHS, staff service; PPI, patient personal issue; HFS, health facility standard; PCA, postoperative patient consciousness of appearance; LRC, likelihood to recommend clinic.

* Sig. at *p* ≤ 0.05

The seven hypotheses that denote direct relation from the element of the patient experience to LRC (H8–H14), interestingly, were only significant in H8, indicating that accessibility (ACS) is associated positively with LRC. H12 referring to staff service (SHS) was found to be significant with a *p* < 0.05 and CI not straddling a zero within the range (negative to negative). However, it has a negative relation, shown by the coefficient −0.157, which is not aligned with the research hypothesis that stated a positive relation; thus, H12 was not supported. On another side, SHS was found to have a positive association with PCA; moreover, it has a greater coefficient compared to other patient experience elements, meaning that SHS is worth paying close attention to.

Ultimately, PCA demonstrated a large effect on LRC (β: 0.403; *p* = 0.004; CI = 0.173–0.671). These findings demonstrate that PCA as a measure of outcome quality can predict LRC adequately. The more patients feel there is a better chance in the consciousness of appearance, the more likely they are to recommend this clinic to others.

Furthermore, an analysis of mediation was also done to determine the significance of mediation through the specific indirect effects, as of the recommendation by Nitzl, Roldan and Cepeda (2016). The result shown in Table 3 indicated the mediation effect of PCA did not straddle a zero in between the CI range, therefore confirming that a mediation occurs for six elements of experience except for ACS which shows the insignificance of mediating effect. This result ascertains that PCA has a pivotal role in mediating patient experience towards the likelihood to recommend the plastic surgery clinic. Patient experience should be associated with the outcome quality first, which manifests in the patient’s appearance consciousness after undergoing plastic surgery before it could be impacting the patient’s intent that supports the clinic’s business performance.

Moving on to the managerial perspective, an IPMA is a useful tool for identifying important indicators from the patient perspective (Ringle & Sarstedt 2016). It could lead to indicators that needed to be prioritised for improvement by the managers of the clinic. This method is based on the total effect for importance and means value for performance. Importance-performance map analysis could be depicted in four quadrants, to plot the indicator’s position on the map, based on horizontal and vertical lines derived from the mean value.

**FIGURE 3:**
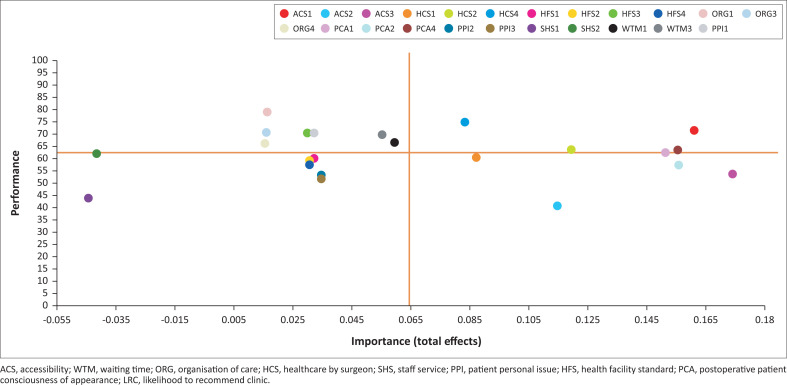
Importance-performance map of indicators.

The IPMA calculation was done by using LRC as the target construct. Figure 3 illustrates the result of the indicators (as in the appendix) to which the clinic management must pay more attention. The indicator on the far right is ACS3 followed by ACS1, where ACS2 is found as the lowest in terms of performance, making it a priority issue. This finding shows that accessibility is the most important for the patient, thus there is an opportunity to manage this problem. Another indicator that is important for patients is the indicator of healthcare by a plastic surgeon (HCS2, HCS1 and HCS4). This finding has sound rationale because plastic surgeons are the most instrumental in the success of a plastic surgery procedure and service. Good communication and trust in the plastic surgeon will make the patient feel worthy of receiving services at the plastic surgery clinic. Therefore, these matters need to be placed as a key factor in the success of plastic surgery.

## Discussion

This research model was developed to find out the contributing factors to the clinic’s likelihood of recommendation (LRC) and whether postoperative PCA serves as a mediator from patient experience. This study analysed patient experience elements, namely, ACS, WTM, ORG, HCS, SHS, PPI and HFS.

The result of this research provides evidence that PCA becomes a relevant construct in understanding how postop patients evaluate the healthcare provider. Patient consciousness of appearance is considered unique and useful because PCA comes from the perspective of patients themselves and not only from the provider’s point of view (Swami et al. 2007), therefore fitting the patient-centred approach. Most outcomes previously used in plastic surgery are psychological in nature, and many of the scales were developed for psychopathology assessment with low content validity (Whalen & Ferrans 2001), including PCA which is derived from the Derriford Scale. This study adds to the health literature with supporting evidence from patient self-report measurement that was validated through empirical study. Although the authors found not all patients show the same degree of emotional responses due to appearance problems, the indicators that are generally applied to a quality-of-life measure for assessing concern about physical appearance need to develop. This study used a unidimensional approach to PCA because it was more convenient for the patient. The result of this study established the construct reliability and validity with three-item questionnaires reflecting PCA (as in the Appendix 1).

The key result of this study provides the evaluation of patient experience elements. Statistical analysis revealed that ACS and HCS had a direct and positive association with LRC (*p* < 0.05), while SHS showed a significant but negative relation (*β* = −0.157). This finding may relate to the staff performance and role in the clinic, albeit this should be confirmed in the larger samples. The role of staff, beginning from the front office and the admission process until the patient leaves the clinic, is crucial. The staff communication through telephone and social media also represents the good communication that patients have with the clinic. Their service will reflect a caring attitude and give an impression. The other five elements of patient experience had a positive association with LRC (*p* < 0.05) mediated by PCA except for ACS. This can be understood because accessibility is tolerable in patients who have frequent visits (Cano et al. 2004). Convenient access to care also relates to external conditions such as traffic and patient domicile. However, the importance of improving patient experience with a clear message regarding accessibility including appointment time needs to be considered.

As expected, PCA demonstrated a large effect on LRC (β: 0.403; *p* = 0.004; CI = 0.173–0.671). Thus, PCA found can mediate five elements of patient experience at the clinic to encourage recommendations from patients to the clinic. This finding is in line with the study from Herruer et al. (2018) that self-consciousness of appearance influences postoperative satisfaction in plastic surgery. This result also aligns with the study from Bellio and Buccoliero (2021) that pointed out that outcome quality leads to behavioural intention. Further, the role of the plastic surgeon (HCS) was found to have a predominant relationship followed by SHS, suggesting that HCS, SHS and ACS should be of utmost concern to plastic surgery patients.

This study obtained from 77% of respondents that the plastic surgeon’s expertise was the reason for choosing the clinic. This is consistent with the result of the study that healthcare provided by the plastic surgeon (HCS) plays an important role. Healthcare service provided by competent and attentive plastic surgeons is the essence of driving patient traffic to the clinic and willingness to recommend the clinic to others. This factor was also found in a previous study (Cogliandro et al. 2016) that evaluated motivation in plastic surgery candidates.

This study found that health facility standards also play a role in impacting PCA. It seems that the more well-equipped the clinic, the more the impression will be positive to the patients. This is in line with the study by Chen et al. (2018) who showed that patients’ convenience in plastic surgery service and their trust in the healthcare provider were also related to the facility. Organisation of care, which includes care coordination with empathy and attention of the physicians, in this case, the plastic surgeons, is highly important in maintaining good doctor–patient relationships. This study found that organisation of care was important from the patient perspective. This study also supports the notion that organisation and documentary record in healthcare providers contribute to achieving good practice, and the electronic medical record is a secure and effective way to deal with this information (Medeiros et al. 2019). Moreover, this organisation of care strategy enables an economy of resources, planning and reduction in patient anxiety because presurgical examinations and assessments are requested for patients with already planned surgery dates.

Another construct that was found to have significant relation was the patient personal issue treated by the clinic. This is in line with a study from Brown et al. (2007) who reported that self-ratings of physical attractiveness predicted a higher likelihood of having aesthetic surgery. This finding supports the notion that failure to attain ideals of attractiveness leads to greater body dissatisfaction and possibly to the consideration of aesthetic surgery to improve appearances (Delinsky 2005). This study is consistent with a previous study from Swami et al. (2007) that personal experience of having had aesthetic surgery was a significant predictor of future likelihood in favour of the healthcare provider. That study confirmed the hypothesis that women would be more likely than men to report willingness to undergo aesthetic surgery (Swami et al. 2007). This is aligned with the current research which acquired 83.6% of female respondents. The difference in gender may play the role in forming a willingness to recommend; thus, gender may moderate the relationship.

This study also revealed that waiting time poses an impact on PCA. Patients seeking aesthetic and reconstructive procedures from plastic surgeons face significant wait times for an initial consultation. Long queuing for appointments and waiting times in clinics have been shown to impede access to adequate healthcare, thereby increasing patient anxiety and potentially decreasing health outcomes. The result of this study is in line with a previous study that evaluates waiting times as a necessary condition for patient satisfaction (Silvestre et al. 2014). The field of plastic surgery is experiencing a workforce shortage due to a stagnant residency training capacity (Salsberg et al. 2008). Specifically, during the pandemic, strategies to reduce the long wait times and increase volume following the pandemic such as COVID-19 will be essential to ensure timely healthcare delivery. This will require strategies to address the growing volume of cases and wait times for surgery across all plastic surgery categories (Saggaf & Anastakis 2021).

The resulting model of this study can denote the explanatory capability of the model (R^2^ and Q^2^) that is meaningful, even though it was found larger for PAC than LRC. Indicating the model could be used to evaluate the plastic surgery clinic as well as gain feedback for management to improve the healthcare service. Patient-reported outcome measurements concerning the quality of life psychologically will provide plastic surgeons with insight to support adequate clinical practice. Nevertheless, this can be useful for the business improvement of the clinic because it will reveal the factors contributing to the likelihood of patient recommendation to the clinic.

## Conclusion

Patient consciousness of appearance as an outcome quality has been proven to mediate patient experience element towards LRC, except for ACS. Further, this study showed a strong association between PCA with the likelihood to recommend the plastic surgery clinic. Reflecting on the results of this study, few managerial implications can be drawn. The clinic management team must focus their attention to optimise HCS, SHS, PCA and all contributing variables impacting PCA. Increasing PCA will also increase the LRC and impact patient traffic and profitability.

Measuring the outcome quality in aesthetic surgery patients is complex. The authors found that the instruments employed in research studies were remarkably diverse, thus yielding difficulties with data collection and analysis with the small sample size. There were also challenges in acquiring responses from the clinic patients because most were aesthetic cases. Aesthetic patients tend to have specific trust issues and a few of them may be non-compliant. Therefore, a study with a larger sample size and homogenous data is required for future studies. PCA’s dimensionality may also be explored in the future with PLS-SEM which enables hierarchical component analysis with a first-order construct.

## Appendix 1

**TABLE 1-A1:** Measurement of patient consciousness of appearance and likelihood to recommend the clinic.

Variables	Indicators
Accessibility (ACS)	Easy for me to book an appointment online or by phone call
	Easy for me to contact the clinic through a phone call, mobile phone, or website/Instagram
	Easy for me to reach the clinic location
Waiting time (WTM)	I feel that waiting time at the clinic for consultation is tolerable
	I feel that the waiting time for the operation schedule is tolerable
Organisation of care (ORG)	I think the clinic already has a clear service guidelines from admission to payment
	I think the clinic already provides information of care with patient needs’ as priority
	I think the clinic already do patient follow-ups well (e.g. next appointment schedule, operation schedule, etc)
Healthcare by surgeon (HCS)	I think the plastic surgeon is communicative and informative to patients
	I think the plastic surgeon has an empathy and understanding towards my personal needs
	I think the plastic surgeon gives the best service to achieve optimal results
Staff service (SHS)	In my opinion, the clinic staffs are very cooperative
	I feel that the clinic staffs are friendly, caring, and polite to the patients
Patient personal issue (PPI)	The clinic service understands my personal needs
	The clinic aware that physical appearance is important for me
	The clinic concern that other people’s opinion about my physical appearance is important
Health facility standard (HFS)	I feel that the clinic environment has standardised cleanliness
	I feel that the equipment and surgical procedures in the consultation and operating room at the clinic are complete and modern
	I feel that the consultation and waiting room at the clinic are well designed
	I feel comfortable at the clinic’s waiting room
Postoperative consciousness of appearance (PCA)	I realise the significant changes of appearance and/or function that I experience after operation
	I feel more confident after undergoing surgery at the clinic
	I feel more comfortable in socialising with my friends after undergoing surgery at the clinic
Likelihood recommendation of clinic (LRC)	I would like to recommend the clinic to others
	I intend to share positive stories about the clinic

## References

[CIT0001] Addo, S.A., Mykletun, R.J. & Olsen, E., 2021, ‘Validation and adjustment of the Patient Experience Questionnaire (PEQ): A regional hospital study in Norway’, *International Journal of Environmental Research and Public Health* 18(13), 7141. 10.3390/ijerph18137141 34281076 PMC8296920

[CIT0002] Bellio, E. & Buccoliero, L., 2021, ‘Main factors affecting perceived quality in healthcare: A patient perspective approach’, *The TQM Journal* 33(7), 176–192. 10.1108/tqm-11-2020-0274

[CIT0003] Brown, A., Furnham, A., Glanville, L. & Swami, V., 2007, ‘Factors that affect the likelihood of undergoing cosmetic surgery’, *Aesthetic Surgery Journal* 27(5), 501–508. 10.1016/j.asj.2007.06.004 19341678

[CIT0004] Cano, S.J., Browne, J.P. & Lamping, D.L., 2004, ‘Patient-based measures of outcome in plastic surgery: Current approaches and future directions’, *British Journal of Plastic Surgery* 57(1), 1–11. 10.1016/j.bjps.2003.08.008 14672672

[CIT0005] Cano, S.J., Klassen, A. & Pusic, A.L., 2019, ‘The science behind quality-of-life measurement: A primer for plastic surgeons’, *Plastic and Reconstructive Surgery* 123(3), 98e–106e. 10.1097/PRS.0b013e31819565c1 19319025

[CIT0006] Carr, T., Harris, D. & James, C., 2000, ‘The Derriford Appearance Scale (DAS-59): A new scale to measure individual responses to living with problems of appearance’, *British Journal of Health Psychology* 5(Part2), 201–215. 10.1348/135910700168865 15969855

[CIT0007] Chen, K., Congiusta, S., Nash, I.S., Coppa, G.F., Smith, M.L., Kasabian, A.K. et al., 2018, ‘Factors influencing patient satisfaction in plastic surgery: A nationwide analysis’, *Plastic and Reconstructive Surgery* 142(3), 820–825. 10.1097/PRS.0000000000004658 30148793

[CIT0008] Chung, K.C., Hamill, J.B., Kim, H.M., Walters, M.R. & Wilkins, E.G., 1999, ‘Predictors of patient satisfaction in an outpatient plastic surgery clinic’, *Annals of Plastic Surgery* 42(1), 56–60. 10.1097/00000637-199901000-00010 9972719

[CIT0009] Coady, M.S., 1997, ‘Measuring outcomes in plastic surgery. Kay-Kilner Prize essay 1996’, *British Journal of Plastic Surgery* 50(3), 200–205. 10.1016/s0007-1226(97)91370-9 9176008

[CIT0010] Cogliandro, A., Persichetti, P., Ghilardi, G., Moss, T.P., Barone, M., Piccinocchi, G. et al., 2016, ‘How to assess appearance distress and motivation in plastic surgery candidates: Italian validation of Derriford Appearance Scale 59 (DAS59)’, *European Review for Medical Pharmacological Sciences* 20(18), 3732–3737. 27735048

[CIT0011] Delinsky, S.S., 2005, Cosmetic surgery: A common and accepted form of self-improvement?’, *Journal of Applied Social Psychology* 35(10), 2012–2028. 10.1111/j.1559-1816.2005.tb02207.x

[CIT0012] Endeshaw, B., 2021, ‘Healthcare service quality-measurement models: A review’, *Journal of Health Research* 35(2), 106–117. 10.1108/jhr-07-2019-0152

[CIT0013] Fenton, J.J., Jerant, A.F., Bertakis, K.D. & Franks, P., 2012, ‘The cost of satisfaction: A national study of patient satisfaction, health care utilization, expenditures, and mortality’, *Archives of Internal Medicine* 172(5), 405–411. 10.1001/archinternmed.2011.1662 22331982

[CIT0014] Hair, J., Risher, J., Sarstedt, M. & Ringle, C., 2019, ‘When to use and how to report the results of PLS-SEM’, *European Business Review* 31(1), 2–24. 10.1108/EBR-11-2018-0203

[CIT0015] Harris, D.L. & Carr, A.T., 2001, ‘The Derriford Appearance Scale (DAS59): A new psychometric scale for the evaluation of patients with disfigurements and aesthetic problems of appearance’, *British Journal of Plastic Surgery* 54(3), 216–222. 10.1054/bjps.2001.3559 11254413

[CIT0016] Henseler, J., Ringle, C. & Sarstedt, M., 2015, ‘A new criterion for assessing discriminant validity in variance-based structural equation modeling’, *Journal of the Academy of Marketing Science* 43(1), 115–135. 10.1007/s11747-014-0403-8

[CIT0017] Herruer, J.M., Prins, J.B., Heerbeek, N., Verhage-damen, G. & Ingels, K., 2018, ‘Does self-consciousness of appearance influence postoperative satisfaction in rhinoplasty?’, *Journal of Plastic, Reconstructive & Aesthetic Surgery* 71(1), 79–84. 10.1016/j.bjps.2017.08.008 28923458

[CIT0018] International Society of Aesthetic Plastic Surgery (ISAPS), Global Survey 2020: Full Report and Press Releases (English), viewed 16 November 2022, from https://www.isaps.org/discover/about-isaps/global-statistics/reports-and-press-releases/global-survey-2020-full-report-and-press-releases-english/

[CIT0019] Liu, S., Li, G., Liu, N. & Hongwei, W., 2021, ‘The impact of patient satisfaction on patient loyalty with the mediating effect of patient trust’, *INQUIRY: The Journal of Health Care Organization, Provision, and Financing* 58, 004695802110072. 10.1177/00469580211007221 PMC8040618 33834860

[CIT0020] Marsidi, N., Maurice, W.H.M. & Luijendijk, R.W., 2014, ‘The best marketing strategy in aesthetic plastic surgery: Evaluating patients’ preferences by conjoint analysis’, *Plastic and Reconstructive Surgery* 133(1), 52–57. 10.1097/01.prs.0000436528.78331.da 24374668

[CIT0021] Medeiros, A.G., Cunha, M.T.R., Tiveron, L.R.C.C., Silva, M.P., Marinho, M.A.O. & Cunha, C.R.R., 2019, ‘Digital organization of plastic surgery service’, *Revista Brasileira de Cirurgia Plástica* 34(4), 517–523. 10.5935/2177-1235.2019RBCP0232

[CIT0022] Memon, M.A., Ramayah, T., Cheah, J.-H., Ting, H., Chuah, F. & Cham, T.H., 2021, ‘PLS- SEM statistical programs: A review’, *Journal of Applied Structural Equation Modeling* 5(1), 1–14. 10.47263/JASEM.5(1)06

[CIT0023] Nitzl, C., Roldan, J.L. & Cepeda, G., 2016, ‘Mediation analysis in partial least squares path modelling: Helping researchers discuss more sophisticated models’, *Industrial Management and Data Systems* 116(9), 1849–1864. 10.1108/IMDS-07-2015-0302

[CIT0024] Pettersen, K.I., 2004, ‘The patient experiences questionnaire: Development, validity and reliability’, *International Journal for Quality in Health Care* 16(6), 453–463. 10.1093/intqhc/mzh074 15557355

[CIT0025] Reavey, P.L., Klassen, A.F., Cano, S.J., McCarthy, C., Scott, A., Rubin, J.P. et al., 2011, ‘Measuring quality of life and patient satisfaction after body contouring: A systematic review of patient-reported outcome measures’, *Aesthetic Surgery Journal* 31(7), 807–813. 10.1177/1090820x11417426 21908812

[CIT0026] Ringle, C.M. & Sarstedt, M., 2016, ‘Gain more insight from your PLS-SEM results the importance-performance map analysis’, *Industrial Management and Data Systems* 116(9), 1865–1886. 10.1108/IMDS-10-2015-0449

[CIT0027] Ringle, C.M., Wende, S. & Becker, J.-M., 2022, *SmartPLS 4*, SmartPLS GmbH, Oststeinbek, viewed 16 November 2022, from http://www.smartpls.com.

[CIT0028] Saggaf, M.M. & Anastakis D.J., 2021, ‘The Impact of COVID-19 on the Surgical wait times for plastic and reconstructive Surgery in Ontario’, *Plastic Surgery* 0(0). 10.1177/22925503211064381 PMC10617460 37915345

[CIT0029] Salsberg, E., Rockey, P.H., Rivers, K.L., Brotherton, S.E. & Jackson, G.R., 2008, ‘US residency training before and after the 1997 Balanced Budget Act’, *Journal of the American Medical Association* 300(10), 1174–1180. 10.1001/jama.300.10.1174 18780846

[CIT0030] Sarstedt, M., Hair, J., Pick, M., Liengaard, B., Radomir, L. & Ringle, C., 2022, ‘Progress in partial least squares structural equation modeling use in marketing research in the last decade’, *Psychology & Marketing* 39(5), 1035–1064. 10.1002/mar.21640

[CIT0031] Shmueli, G., Sarstedt, M., Hair, J., Cheah, J., Ting, H., Vaithilingam, S. et al., 2019, ‘Predictive model assessment in PLS-SEM: Guidelines for using PLS predict’, *European Journal of Marketing* 53(11), 2322–2347. 10.1108/EJM-02-2019-0189

[CIT0032] Silvestre, J., Bess, C.R., Nguyen, J.T., Ibrahim, A.M., Patel, P.P. & Lee, B.T., 2014, ‘Evaluation of wait times for patients seeking cosmetic and reconstructive breast surgery’, *Annals of Plastic Surgery* 73(1), 16–18. 10.1097/SAP.0b013e318276d902 24918733

[CIT0033] Strumann, C., Geissler, A., Busse, R. & Pross, C., 2022, ‘Can competition improve hospital quality of care? A difference-in-differences approach to evaluate the effect of increasing quality transparency on hospital quality’, *The European Journal of Health Economics* 23, 1229–1242. 10.1007/s10198-021-01423-9 34997865 PMC9395484

[CIT0034] Swain, S. & Kar, N.C., 2018, ‘Hospital service quality as antecedent of patient satisfaction – A conceptual framework’, *International Journal of Pharmaceutical and Healthcare Marketing* 12(3), 251–269. 10.1108/IJPHM-06-2016-0028

[CIT0035] Swami, V., Arteche, A., Chamorro-Premuzic, T., Furnham, A., Stieger, S., Haubner, T. et al., 2007, ‘Looking good: Factors affecting the likelihood of having cosmetic surgery’, *European Journal of Plastic Surgery* 30(5), 211–218. 10.1007/s00238-007-0185-z

[CIT0036] Wolf, J.A., Niederhauser, V., Marshburn, D. & LaVela, S.L., 2014, ‘Defining patient experience’, *Patient Experience Journal* 1(1), 7–19. 10.35680/2372-0247.1000

[CIT0037] Whalen, G.F. & Ferrans, C.E., 2001, ‘Quality of life as an outcome in clinical trials and cancer care: A primer for surgeons’, *Journal of Surgical Oncology* 77(4), 270–276. 10.1002/jso.1107 11473376

[CIT0038] Yeo, S.F., Tan, C.L. & Goh, Y.-N., 2021, ‘Obstetrics services in Malaysia: Factors influencing patient loyalty’, *International Journal of Pharmaceutical and Healthcare Marketing* 15(3), 389–409. 10.1108/ijphm-08-2020-0070

